# Case Report: Myelodysplastic changes and 7q deletion in a case of vitamin B12 deficiency: a diagnostic pitfall

**DOI:** 10.3389/fnut.2026.1883509

**Published:** 2026-10-01

**Authors:** Ritika Khurana, Purva Kanvinde, Sangeeta Mudaliar

**Affiliations:** Department of Paediatric Hematology-Oncology, Bai Jerbai Wadia Hospital for Children, Mumbai, Maharashtra, India

**Keywords:** dyserythropoiesis, myelodyslastic syndromes, nutritional deficiences, pancytopenia, vitamin B12

## Abstract

Vitamin B12 plays a crucial role in DNA synthesis, and its deficiency can occasionally present with atypical hematological manifestations mimicking myelodysplastic syndrome (MDS). There is limited literature describing reversible chromosomal abnormalities associated with vitamin B12 deficiency. We report an 11-year-old girl who presented with generalized body aches for 1 month and fever, easy fatigability, and respiratory distress for 2 days. Examination revealed pallor and hepatomegaly, with no splenomegaly. Investigations showed severe anemia (hemoglobin 3.1 g/dL), thrombocytopenia, a low corrected reticulocyte count, and macrocytosis with macro-ovalocytes on a peripheral smear. Considering pancytopenia with congestive cardiac failure, the differential diagnoses included viral infection-induced cytopenias, leukemia, megaloblastic anemia, and MDS. Bone marrow examination demonstrated myelodysplastic changes, while cytogenetic analysis revealed a 7q deletion in 18% of metaphases. Serum vitamin B12 levels were markedly low (<83 pg./mL). The child received a packed red cell transfusion and oral vitamin B12 supplementation, following which the blood counts improved significantly. Repeat bone marrow examination after 2 months showed complete resolution of the abnormalities. Vitamin B12 deficiency can rarely lead to chromosomal abnormalities such as 7q and 20q deletions, aneuploidy, and hypoploidy because of impaired DNA synthesis and uracil misincorporation into DNA strands. Such abnormalities are classically associated with MDS and acute myeloid leukemia, potentially leading to diagnostic confusion and unnecessary aggressive treatment. Recognition of vitamin B12 deficiency as a reversible cause of dysplastic marrow changes and cytogenetic abnormalities is therefore essential. Early identification and prompt treatment can result in complete hematological and cytogenetic recovery.

## Introduction

Vitamin B12 plays a vital role in DNA synthesis. There is limited literature describing chromosomal aberrations in patients presenting with features suggestive of myelodysplastic syndrome (MDS) who are subsequently found to have underlying vitamin B12 deficiency. The 7q deletion and a few other abnormalities, such as 20q deletion, arise from impaired DNA synthesis and can be reversed with vitamin B12 supplementation (1).

## Case description

An 11-year-old girl presented with generalized body aches for 1 month, along with fever, easy fatigability, and respiratory distress for 2 days. There was no significant medical history. The child had been consuming a vegetarian diet, and there was no relevant family history of transfusions or jaundice. On examination, vital signs and anthropometric measurements were normal. She was pale, with hepatomegaly (liver palpable 5 cm below the costal margin) and no splenomegaly. Table 1 presents the baseline investigations.

**Table 1:** Baseline investigations.

Investigations	Findings
Hemoglobin	3.1 g/dL
MCV/MCH/MCHC	98 fL/35 pg./33 g/dL
Reticulocyte count	2.6% (corrected, 0.6%)
White blood cell count	1.9 × 10^9^/L
Differential count (ANC, ALC)	1.08 × 10^9^/L, 0.74 × 10^9^/L
Platelet count	99,000/μL
Peripheral smear	Macrocytes with macro-ovalocytes; normal WBC morphology; adequate platelets

ALC, absolute lymphocyte count; ANC, absolute neutrophil count; MCH, mean corpuscular hemoglobin; MCHC, mean corpuscular hemoglobin concentration; MCV, mean corpuscular volume; WBC, white blood cell count.

In view of the acute febrile illness with pancytopenia and congestive cardiac failure, the differential diagnoses considered included viral infection-induced cytopenias, leukemia, megaloblastic anemia, and myelodysplastic syndrome. Bone marrow examination revealed increased cellularity with erythroid hyperplasia, megaloblastic changes, and dyserythropoiesis; the granulocytic series was suppressed, with adequate megakaryopoiesis. Conventional karyotyping revealed an interstitial del (7q) in 36/200 metaphases (18%): [ISCN]. Serum vitamin B12 levels were less than 83 pg./mL. Myeloid next-generation sequencing was not performed because of financial constraints. The child received a packed red cell transfusion and was started on oral vitamin B12 (methylcobalamin), 750 μg daily. Within 1 week, the cytopenias began to improve, with near normalization by week 3 (Figure 1). As part of further evaluation, parietal cell antibody testing was weakly positive, while thyroid function test results were normal. Figure 1 shows the complete blood count (CBC) trend.

**Figure 1:**
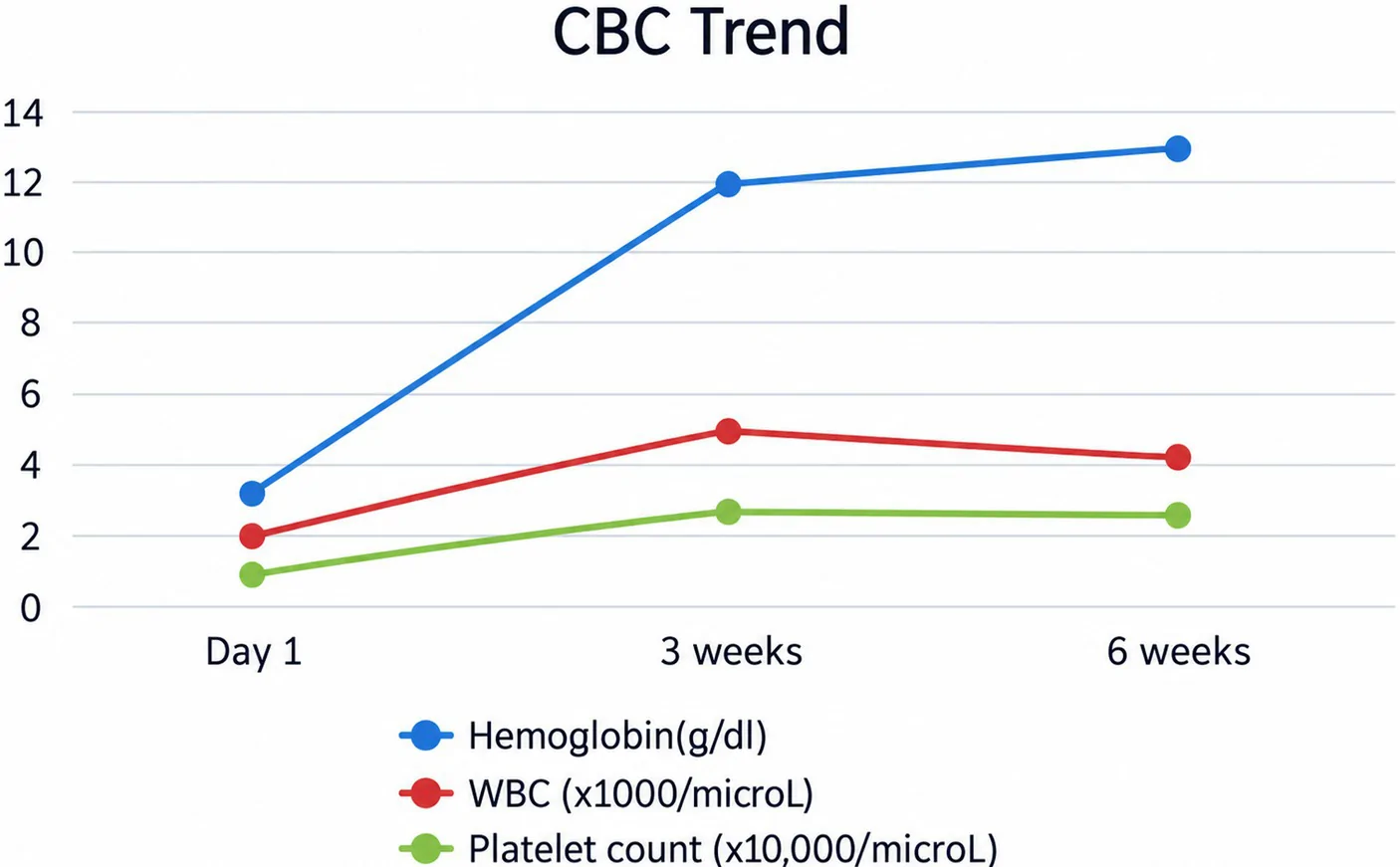
Complete blood count (CBC) trends over 6 weeks of treatment.

A repeat bone marrow examination showed normocellular marrow with trilineage hematopoiesis, no dysplasia, <5% blasts, and no 7q deletion on cytogenetic evaluation.

## Discussion

The classic presentation of vitamin B12 deficiency includes anemia and, occasionally, neurological manifestations. Investigations show macrocytosis, hypersegmented neutrophils, and low vitamin B12 levels. However, children presenting with atypical features, such as the involvement of multiple cell lines or organomegaly, may require bone marrow examination.

MDS comprises a heterogeneous group of disorders characterized by dysplastic and ineffective hematopoiesis, with a risk of progression to leukemia. Bone marrow findings in vitamin B12 deficiency may closely resemble those seen in MDS, as both conditions can result in ineffective hematopoiesis and bone marrow failure. Deletion of 7q is most commonly associated with MDS or acute myeloid leukemia (AML), particularly therapy-related AML.

Chromosomal abnormalities have been reported in patients with vitamin B12 deficiency. One proposed mechanism is that vitamin B12 deficiency leads to the accumulation of homocysteine and folate trapping, resulting in increased deoxyuridine monophosphate (dUMP), which interferes with the incorporation of deoxythymidine monophosphate (dTMP) into DNA. A high intracellular dUMP/dTMP ratio and a subsequent increase in the deoxyuridine triphosphate/deoxythymidine triphosphate (dUTP/dTTP) ratio result in the misincorporation of uracil into DNA strands (2, 3). A few pediatric case reports have been published, but some reports describe adults presenting with macrocytic anemia and bone marrow findings suggestive of myelodysplasia with a 7q deletion. In one such case, erythropoietin therapy was initially administered without improvement. Vitamin B12 therapy was subsequently initiated, after which the patient improved (4). Reported abnormalities include hypoploidy, aneuploidy, 3p deletions, and 20q deletions. All reported cases demonstrated reversal following vitamin B12 replacement, with some showing reversal within 48 h (1, 5).

## Conclusion

Cytogenetic abnormalities such as 7q and 20q deletions are often recognized as precursors to myeloid neoplasms such as AML. In patients with pancytopenia and macrocytosis, vitamin B12 levels should be measured (along with folate, methylmalonic acid [MMA], and homocysteine) before the condition is labeled as MDS. If cytogenetic abnormalities are present, consider a therapeutic trial of vitamin B12 supplementation with close monitoring before initiating disease-modifying MDS therapy in appropriate clinical contexts.

## Data Availability

The datasets presented in this article are not readily available because of ethical and privacy restrictions. Requests to access the datasets should be directed to the corresponding author.
